# Rice Germ Ameliorated Chronic Unpredictable Mild Stress-Induced Depressive-like Behavior by Reducing Neuroinflammation

**DOI:** 10.3390/nu14245382

**Published:** 2022-12-18

**Authors:** Sosorburam Batsukh, Seyeon Oh, Kyoungmin Rheu, Bae-Jin Lee, Chul-Hyun Park, Kuk Hui Son, Kyunghee Byun

**Affiliations:** 1 Department of Anatomy & Cell Biology, College of Medicine, Gachon University, Incheon 21936, Republic of Korea; 2 Functional Cellular Networks Laboratory, Lee Gil Ya Cancer and Diabetes Institute, College of Medicine, Gachon University, Incheon 21999, Republic of Korea; 3 Marine Bioprocess Co., Ltd., Smart Marine BioCenter, Gijang-gun, Busan 46048, Republic of Korea; 4 Department of Thoracic and Cardiovascular Surgery, Gachon University Gil Medical Center, Gachon University, Incheon 21565, Republic of Korea

**Keywords:** rice germ, gamma-aminobutyric acid, chronic unpredictable mild stress, hypothalamic neuroinflammation, pyroptosis

## Abstract

Stress-induced neuroinflammation is widely regarded as one of the primary causes of depression. Gamma-aminobutyric acid (GABA)-enriched foods relieve stress and reduce inflammatory reactions. This study aimed to evaluate whether rice germ with 30% GABA (RG) reduced neuroinflammation in mice exposed to chronic unpredictable mild stress (CUMS). CUMS mice were administered 40, 90, and 140 mg/kg of RG. CUMS increased serum and hypothalamic pro-inflammatory cytokine (TNF-α and IL-6) levels, which were decreased by RG. In the hypothalamus, CUMS elevated M1-type microglia markers of CD86 and NF-κB, whereas RG lowered these levels. The expression levels of NLRP3 inflammasome complex (NLRP3, apoptosis-associated speck-like protein containing a caspase recruitment domain, and caspase-1), IL-1β, and IL-18 were increased in the hypothalamus of CUMS mice and decreased by RG. RG attenuated depressive-like behaviors in CUMS mice, as measured by the forced swim test and tail suspension test. In conclusion, RG decreased hypothalamic inflammation-related signals, such as TNF-α, IL-6, M1 polarization, NF-κB, NLRP3 inflammasome complex, caspase-1, IL-1β, and IL-18, to diminish depressive-like behavior.

## 1. Introduction

Stress causes aberrant regulation or malfunction of the hypothalamic-pituitary-adrenal (HPA) axis, resulting in cognitive impairment, depression, and anxiety [1,2]. Stress-induced neuroinflammation is considered one of the primary causes of depression. The expression levels of pro-inflammatory cytokines, such as tumor necrosis factor alpha (TNF-α), interleukin (IL)-6 and IL-1β have been found to be elevated in the peripheral blood or central nervous system (CNS) of patients suffering from major depressive disorder (MDD) [3,4]. Furthermore, these pro-inflammatory cytokines were found to be elevated in numerous animal models of stress, and increased pro-inflammatory cytokines are associated with depressive-like behaviors [5,6,7,8].

Microglia initiate neuroinflammation during depression. Microglia can polarize to-ward the pro-inflammatory M1-type, resulting in NF-κB upregulation and increased TNF-α, IL-1β, and IL-6 production [9,10]. M1 can upregulate IL-6 and TNF-α; however, IL-6 and TNF-α also promote M1 polarization [11,12,13]. M1 and NF-κB lead to an increased level of NOD-like receptor family pyrin domain containing 3 (NLRP3) inflammasome complex [14,15,16,17,18]. The NLRP3 inflammasome complex induces pyroptosis, a form of cell death mediated by gasdermins (GSDMDs) [19]. The NLRP3 inflammasome complex converts pro-caspase-1 into an active form of caspase-1, which can cleave pro-IL-1β and pro-IL-18 and generate GSDMD-induced cell membrane pores [19,20]. The pores disrupt the cell membrane, resulting in pyroptosis and leakage of the pro-inflammatory cytokines such as IL-1β and IL-18 [21,22].

Recently, gamma-aminobutyric acid (GABA)-enriched foods, such as rice or tea, have been reported to reduce stress in humans [23,24]. GABA-enriched rice consumption attenuates anxiety scores and blood cortisol levels [24]. GABA occurs naturally in different types of food, particularly vegetables and grains, such as rice germ [25]. Rice germ is a grain fraction rich in protein content, essential amino acids, fatty acids, and fiber [26]. Fermentation process also increased GABA content in various natural source. Previous studies have shown that fermentation with *Lactobacillus brevis* BJ 20 can significantly increase GABA levels in oyster extract and seaweed [27,28].

Since GABA cannot be delivered directly to the CNS across the blood–brain barrier (BBB) [29,30], the specific mechanism by which GABA supplements reduce stress is debatable [31]. In lipopolysaccharide-stimulated RAW 264.7 cells, GABA showed anti-inflammatory activity by decreasing the levels of IL-1β, inducible nitric oxide synthase, and TNF-α [32]. GABA decreases the expression level of pro-inflammatory cytokines and NF-κB activity in lymphocytes [33]. Increased expression level of pro-inflammatory cytokines in the peripheral blood could affect the CNS since they can cross the BBB [34].

Although previous studies have shown that GABA can attenuate stress-related dis-orders, it has not been revealed whether rice germ with 30% GABA (RG) can attenuate stress-induced depression by reducing neuroinflammation in the brain.

Thus, the purpose of our study was to evaluate whether RG decreased stress-related disorder via decreasing neuroinflammation which induced by increased pro-inflammatory cytokine and increased NLRP3 inflammasome.

We hypothesized that RG decreased the levels of peripheral pro-inflammatory cytokines, such as TNF-α and IL-6 eventually decreasing the levels of TNF-α and IL-6 in the brain. Decreased pro-inflammatory cytokine levels reduced M1 polarization and NF-κB expression, subsequently decreasing the level of the NLRP3 inflammasome complex. Reduced neuroinflammation leads to a decrease in stress-induced depressive behavior. We evaluated our hypothesis using a chronic unpredictable mild stress (CUMS) mice model. L-theanine, which is known to reduce stress-induced depression [35], was used as a positive control to evaluate the stress-reducing effect of RG.

## 2. Materials and Methods

### 2.1. Preparation of RG

RG was acquired from Marine Bioprocess Co., Ltd. (Busan, Republic of Korea). The rice germ purchased from Hwasung Energy Co. Ltd. (Gyeongsan, Republic of Korea) was manually washed three times with water and drained. Rinsed rice germ was mixed with water at a ratio of 1: 10, and then sprayed with 1% amylase (Ban^®^ 480 L, FG, Novozyms Korea Co., Ltd., Seoul, Republic of Korea) and hydrolyzed at 67 ± 2 °C for 4 h. After hydrolysis, rice germ was autoclaved at 90 °C for 30 min and then at 121 °C for 10 min to ensure inactivation of amylase.

The hydrolyzed rice germ was filtered with a vibrating sieve (120 mesh, BÜCHI Labortechnik GmbH, Essen, Germany) and concentrated by rotary evaporator (BÜCHI Labortechnik GmbH).

After sterilizing the seed medium composed of yeast extract 3%, glucose 1%, monosodium glutamate 1%, and water 95% for 15 min at 121 °C, Lactobacillus brevis BJ20 (accession No. KCTC 11377BP) was inoculated and cultured for 24 h at 37 °C. Then, 10% (*v*/*v*) of the *Lactobacillus brevis BJ20* cultured seed medium was fermented in a fermentation medium (yeast extract 1.5%, glucose 0.5%, monosodium glutamate 8%, L-glutamic acid 24%, hydrolyzed rice germ extracts 50%, water 16%) at 37 °C for 72 h. The fermentation medium was filtered (disk separator, CLARA 200, Alfa Laval, Lund, Sweden), dextrin supplemented (Matsutani Korea Co. Ltd. Bundang, Republic of Korea), and spray-dried to prepare rice germ powder samples.

### 2.2. High-Performance Liquid Chromatography (HPLC) Analysis

#### 2.2.1. Chemical and Reagent

GABA and sodium acetate (50 mM, pH 6.5) were manufactured by Sigma-Aldrich (St. Louis, MO, USA). HPLC-grade acetonitrile, methanol, and distilled water (DW) were manufactured by Samchun Pure Chemical Co. Ltd. (Pyeongtaek, Republic of Korea). The hydrochloric acid solution was manufactured by Biosesang (Seongnam, Republic of Korea). Borate buffer (0.4 N in water, pH 10.2; Agilent P/N 5061-3339) and o-Phthaldialdehyde reagent (10 mg/mL, Agilent P/N 5061-3335) were manufactured by Agilent Technologies (Palo Alto, CA, USA). Acetic acid was manufactured by Junsei Chemical Co. Ltd. (Tokyo, Japan).

#### 2.2.2. Standard Solution and Sample Preparation

A 0.1 g standard sample was dissolved in 100 mL of DW in a volumetric flask to prepare a standard solution, filtered through a polytetrafluoroethylene syringe filter (25 mm/0.2 μm), and kept at −80 °C. For the 5% aqueous sample solution, 5 g of RG was dissolved in DW in a 100 mL volumetric flask and filtered using a polytetrafluoroethylene syringe filter.

#### 2.2.3. HPLC Analysis Method

GABA content was determined using the method described [36], with minor changes. In our study, a Dionex U3000 series HPLC system (Thermo Fisher, Waltham, MA, USA) equipped with a UV detector was utilized, and the flow rate was lowered to 1 mL/min. The samples were analyzed using UV–Vis spectrophotometry at a wavelength of 338 nm. The amount of GABA in the RG was calculated using the following Equation:

Substance (mg/g) = Measurement (mg/mL) × Dilution factor ÷ Amount(g) × 100 (mL)

### 2.3. Animals and CUMS Procedure

C57BL/6 (Male) mice were provided by Orient Bio (Seongnam, Republic of Korea). The animals were bred under standard conditions (23 ± 2 °C, 12 h light/dark cycle, humidity of 50 ± 3%). All animal experiments were conducted in accordance with the ethical principles of the Institutional Animal Care and Use Committee of Gachon University (approval No. LCDI-2021-0170).

After an acclimatization period of 1 week, the CUMS procedure was conducted for 5 weeks. In our study, 24 h of food deprivation, rein for 24 h in an empty water bottle, exposure to foreign object for 24 h and wet bedding for 24 h were a stressor (Figure S1A).

### 2.4. Animal Experimental Design

The mice were randomly separated into seven groups (*n* = 6):

(1) Control: The group was administered orally the same volume of saline as that administered in the other groups without the CUMS procedure.
(2) CUMS: The group was administered orally the same volume of saline as that administered in the CUMS procedure.
(3) CUMS/RG40: The group was administered orally RG at 40 mg/kg daily with the CUMS procedure.
(4) CUMS/RG90: The group was administered orally RG at 90 mg/kg daily with the CUMS procedure.
(5) CUMS/RG140: The group was administered orally RG at 140 mg/kg daily with the CUMS procedure.
(6) CUMS/GABA: The group was administered orally GABA at 30 mg/kg daily with the CUMS procedure.
(7) CUMS/Theanine: The group was administered orally theanine at 50 mg/kg daily with the CUMS procedure.

After the CUMS procedure for 5 weeks, RG, GABA, and theanine were administered orally at the same time as the CUMS procedure over 4 weeks.

Behavioral tests were performed at the last oral administration, and the mice were sacrificed under respiratory anesthesia with isoflurane (Figure S1B). Blood and brain samples were collected for this study.

### 2.5. Forced Swimming Test (FST)

FST was performed with slight changes based on what was described in previous study [37,38]. Briefly, before the experiment, an open cylindrical container with a height of 45 cm and a diameter of 25 cm was filed water at 26 ± 1 °C to a depth of 35 cm.

The experimenter holds the mice by the tail and slowly placed the mouse into the container with water. The mice were adapted to the water for 15 min and the main test was performed after 24 h. After filling the open cylindrical container with water under the same conditions as the previous day, the mice were placed in the water and FST was performed for 7 min. During the FST, the experimenter observed while maintaining an appropriate distance, and when the mouse submerged, it was removed from the container. After 7 min of FST, the recording was stopped, and the mice were taken out of the water, gently dried with dry paper, and put back into the cage.

For FST analysis, only the last 5 min of the 7 min-FST were analyzed. This is because most mice were active at the beginning of the FST, so the potential effects of the treatment may be masked during the first 2 min. Immobility time was analyzed using Smart 3.0. program (PanLab Harvard Apparatus, MA, USA).

### 2.6. Tail-Suspension Test (TST)

TST was conducted with minor modifications, referring to the content described in previous study [39]. Briefly, the tail of the mice was hung upside down in a suspension box (45 cm height, 25 cm diameter). After the tail was wrapped with tape, the head of each mouse was approximately 20 cm from the floor. And TST was performed for 6 min. After 6 min of TST, the recording was stopped, and the mice were placed in a cage with the tape on its tail removed.

For TST analysis, last 4 min of the suspension time of 6 min was analyzed. This is due to most mice were active during first 2 min. Immobility time was measured and analyzed using Smart 3.0. program (PanLab Harvard Apparatus).

### 2.7. Enzyme-Linked Immunosorbent Assay

To measure the expression levels of TNF-α and IL-6 in the serum, collected blood was placed in a serum separator tubes (Becton Dickinson, Franklin Lakes, NJ, USA) and incubated at room temperature for 20 min. Thereafter, the blood samples were centrifuged at 2000× *g* for 20 min, and the supernatant was separated and put to a new tube.

The 96-well microplates coated with a coating buffer (pH 9.6) containing 100 nM sodium carbonate and sodium bicarbonate was incubated with 5% skim milk at room temperature for 2 h to block unnecessary protein binding. After rinsing with phosphate buffered saline containing tween-20 (TPBS), equal amounts (100 μg) of samples were placed in each well and incubated 12 h at 4 °C. And then washed with TPBS and incubated for 12 h at 4 °C with appropriate aliquots of anti-TNF-α and anti-IL-6 antibodies (Table S1), respectively. Thereafter, each well was washed again and incubated for 2 h at room temperature with peroxidase-conjugated antibody. After rinsing with TPBS, a tetramethylbenzidine solution (100 μL/well; Sigma-Aldrich) was added for color development, and incubation was performed at room temperature for 15–20 min while blocking the light. After color development, the same volume of stop solution (100 μL/well; sulfuric acid, 2N) was dispensed, and optical density was analyzed at a wavelength of 450 nm using a Multiskan SkyHigh Microplate Spectrometer (ThermoFisher Scientific, Waltham, MA, USA).

### 2.8. 3,3′-Diaminobenzidine (DAB) Staining

The brain tissue of mice was fixed in cold 4% paraformaldehyde (Sigma-Aldrich) for 4 h. Paraffin-embedded tissue blocks were prepared using a tissue processor (Tissue-Tek VIP^®^ 5 Jr, SAKURA Finetek, Tokyo, Japan) and an embedding machine (Tissue-Tek^®^ TEC™ 6, SAKURA Finetek) from fixed brain tissue. The paraffin-embedded brain blocks were cut into 5 µm thickness using a microtome (ThermoFisher Scientific, Waltham, MA, USA) and dried at 60 °C for 24 h to adhere well to slides. Before starting DAB staining, paraffin was removed from the slides and the tissues were boiled in the sodium citrate buffer (pH 6.0) using a microwave oven, and then cooled in cold distilled water for antigen retrieval. To prevent non-specific antibody binding, after incubation in normal serum (Vector Laboratories, Burlingame, CA, USA) for 1 h, appropriate concentration of primary antibodies in normal serum (Table S1) was incubated followed by rinsing with PBS.

Then, the primary antibodies-tagged brain tissues were incubated with biotinylated secondary antibodies (Vector Laboratories) and washed with PBS. For brown color development, the slides were reacted with DAB solution activated with H_2_O_2_ for 15 min. The stained tissues were mounted with coverslip and DPX solution (Sigma-Aldrich). Images were obtained using a slide scanner (Motic, Kowloon, Hong Kong), and the brown color intensity was analyzed with ImageJ software (NIH, Bethesda, MD, USA).

### 2.9. Quantitative Real-Time Polymerase Chain Reaction (qRT-PCR)

RNA of the hypothalamus was isolated using RNAiso (Takara, Tokyo, Japan) according to the manufacturer’s instructions. The extracted RNA was synthesized with cDNA using a PrimeScript first-strand cDNA Synthesis Kit (Takara). qRT-PCR was performed using cDNA synthesized using the CFX384 TouchTM Real-Time PCR detection system. cDNA (300 ng), SYBR premix (5 μL; Takara), forward primer (0.4 μM), and reverse primer (0.4 μM) were mixed, and the number of threshold cycle were determined using CFX ManagerTM software (BioRad, CA, USA). All primer information is summarized in the Table S2.

### 2.10. Statistical Analysis

Non-parametric tests were conducted in this study. The Kruskal–Wallis test was used to confirm the importance of differences between the seven groups. Multiple comparisons were performed using Mann–Whitney U test if significant difference were found in the Kruskal–Wallis test. Results were expressed as mean ± standard deviation, and statistical significance was accepted as follows: * versus Non-CUMS; $ versus CUMS; # versus CUMS/RG90; † versus CUMS/GABA; ^ versus CUMS/Theanine. Statistical analyses were performed using SPSS version 22 (IBM Corporation, Armonk, NY, USA).

## 3. Results

### 3.1. GABA Content in RG

To determine the GABA content in RG, we performed HPLC analysis. The presence of GABA after fermentation was confirmed by comparing the retention times of the standard and RG (Figure S2 and Table S3). Furthermore, HPLC chromatography showed that the average percent content of GABA in RG was 31.04 ± 0.94%.

### 3.2. RG Decreased Expression Levels of TNF-α and IL-6 in Serum and Hypothalamic of CUMS Mice

First, we evaluated serum IL-6 and TNF-α levels in control, CUMS, CUMS/RG40, CUMS/RG90, CUMS/RG140, CUMS/GABA, and CUMS/Theanine groups. When RG 40 mg/kg was administered to the animal, the GABA content consumed by the animal was 12 mg/kg. When RG 90 and 140 mg/kg were administered to animal, the content of GABA which animal were consumed were 27 mg/kg and 42 mg/kg, respectively.

**Figure 1:**
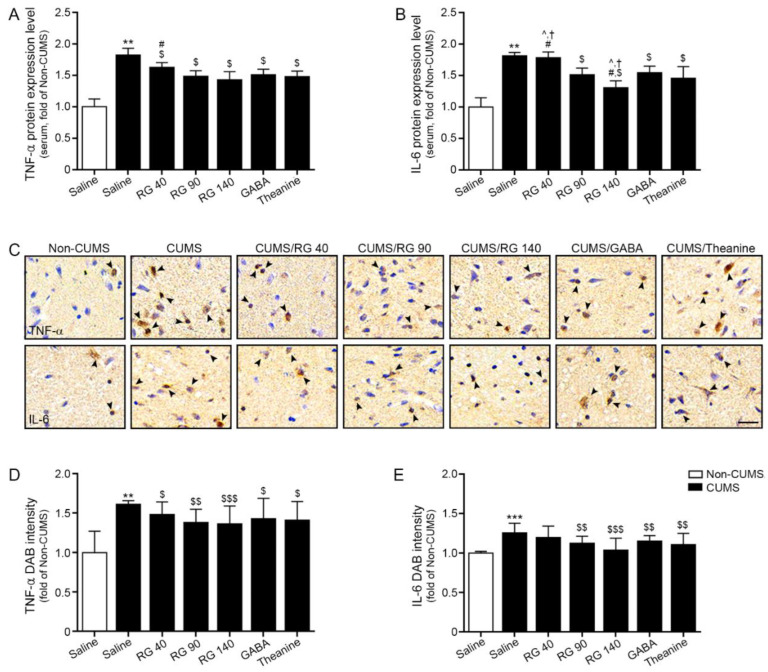
Reduction effect of RG on TNF-α and IL-6 expression levels in the serum and hypothalamus of CUMS mice model. (**A**,**B**) TNF-α (**A**) and IL-6 (**B**) protein expression levels were significantly increased in serum of CUMS compared to those in the Non-CUMS group and decreased upon RG, GABA, and theanine treatment in CUMS; (**C**–**E**) TNF-α (**upper lane of** (**C**)) and IL-6 (**lower lane of** (**C**)) expression levels in the hypothalamus of CUMS were validated using DAB staining (**C**, scale bar = 200 μm). Arrows point positive signals. Quantification graphs (**D**,**E**) are depicted in (**C**). The expression levels of TNF-α and IL-6 were higher in CUMS compared to those in control. However, their expression was decreased after RG, GABA, and theanine treatment. Data are presented as the mean ± SD (*n* = 3/group). CUMS, chronic unpredictable mild stress; DAB, 3,3′-diaminobenzidine; GABA, gamma-aminobutyric acid; IL-6, interleukin-6; RG, rice germ with 30% GABA; TNF-α, tumor necrotic factor-alpha. **, *p* < 0.01 and ***, *p* < 0.05 vs. Non-CUMS; $, *p* < 0.05, $$, *p* < 0.01 and, $$$, *p* < 0.001 vs. CUMS; #, *p* < 0.05 vs. CUMS/RG90; †, *p* < 0.05 vs. CUMS/GABA; ^, *p* < 0.05 vs. CUMS/Theanine.

The serum TNF-α levels were significantly increased by CUMS. It was decreased by oral administration of RG 40 mg/kg (10.8% decreased compared with CUMS group), RG 90 mg/kg (18.5% decreased compared to CUMS group), and 140 mg/kg (21.6% decreased compared to CUMS group), GABA (17.2% decreased compared to CUMS group), and theanine (18.8% decreased compared with CUMS group). The reduction effects of RG (90 and 140 mg/kg), GABA, and theanine were not significantly different (Figure 1A).

The serum IL-6 level was significantly increased by CUMS. It was decreased by oral administration of RG (90 and 140 mg/kg), GABA, and theanine. This reduction was most prominent at RG 140 mg/kg (Figure 1B).

TNF-α expression in the hypothalamus increased significantly after CUMS treatment. It was decreased by oral administration of RG (40, 90, and 140 mg/kg), GABA, and theanine. The reduction effects of RG (40, 90, and 140 mg/kg), GABA, and theanine did not differ significantly (Figure 1C,D).

CUMS significantly increased the hypothalamic IL-6 expression. It was decreased by the administration of RG (90 and 140 mg/kg), GABA, and theanine. The reduction effects of RG (90 and 140 mg/kg), GABA, and theanine did not differ significantly (Figure 1C,E).

### 3.3. RG Decreased M1 Polarization and NF-κB Expression

**Figure 2:**
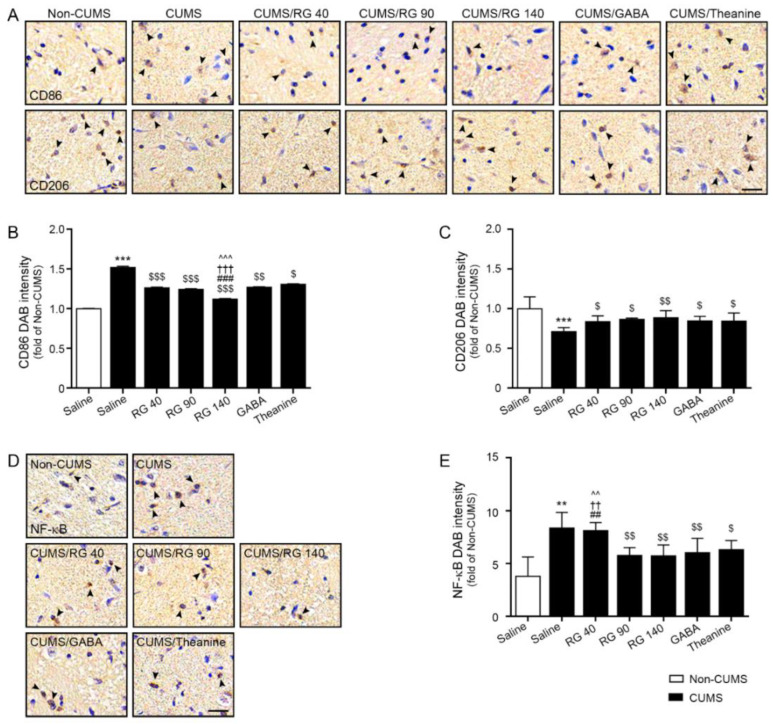
Effect of NF-κB reduction due to M1 reduction and M2 elevation by RG in the hypothalamus of CUMS mice model. (**A**) The expression levels of CD86 (M1 marker; **upper lane in** (**A**)), CD206 (M2 marker; **lower lane in** (**A**)), and NF-κB (**D**) in the hypothalamus of CUMS were validated using DAB staining (scale bar = 200 μm). Arrows point positive signals; (**B**,**C**,**E**) Quantification graphs are depicted in (**A**,**D**). The expression levels of CD86 (**B**) and NF-κB (**E**) increased in CUMS relative to those in the control. However, their expression decreased after RG, GABA, and theanine treatment. CD206 (**C**) expression decreased in CUMS compared to that in the Non-CUMS. However, their expression increased after RG, GABA, and theanine treatment. Data are presented as the mean ± SD (*n* = 3/group). CD206, cluster of differentiation 206; CD86, cluster of differentiation 86; CUMS, chronic unpredictable mild stress; DAB, 3,3′-diaminobenzidine; GABA, gamma-aminobutyric acid; RG, rice germ with 30% GABA; NF-κB, nuclear factor kappa-light-chain-enhancer of activated B cells. **, *p* < 0.01 and ***, *p* < 0.05 vs. Non-CUMS; $, *p* < 0.05, $$, *p* < 0.01 and, $$$, *p* < 0.001 vs. CUMS; ##, *p* < 0.01 and ###, *p* < 0.001 vs. CUMS/RG90; ††, *p* < 0.01 and †††, *p* < 0.001 vs. CUMS/GABA; ^^, *p* < 0.01 and ^^^, *p* < 0.001 vs. CUMS/Theanine.

CUMS significantly increased CD86 (M1 marker) expression in the hypothalamus. It was decreased by oral administration of RG (40, 90, and 140 mg/kg), GABA, and theanine. This reduction was most prominent with RG at 140 mg/kg (Figure 2A,B).

CUMS significantly decreased CD206 (M2 marker) expression. It was increased by the administration of RG (40, 90, and 140 mg/kg), GABA, and theanine. The reduction effects of RG (40, 90, and 140 mg/kg), GABA, and theanine did not differ significantly (Figure 2A,C).

CUMS significantly increased the expression of NF-κB in the hypothalamus. It was decreased by oral administration of RG (90 and 140 mg/kg), GABA, and theanine. The effects of RG (90 and 140 mg/kg), GABA, and theanine on NF-κB expression did not differ significantly (Figure 2D,E).

### 3.4. RG Decreased the Expression Levels of the NLRP3 Inflammasome Complex, Caspase-1, IL-1β, and IL-18

**Figure 3:**
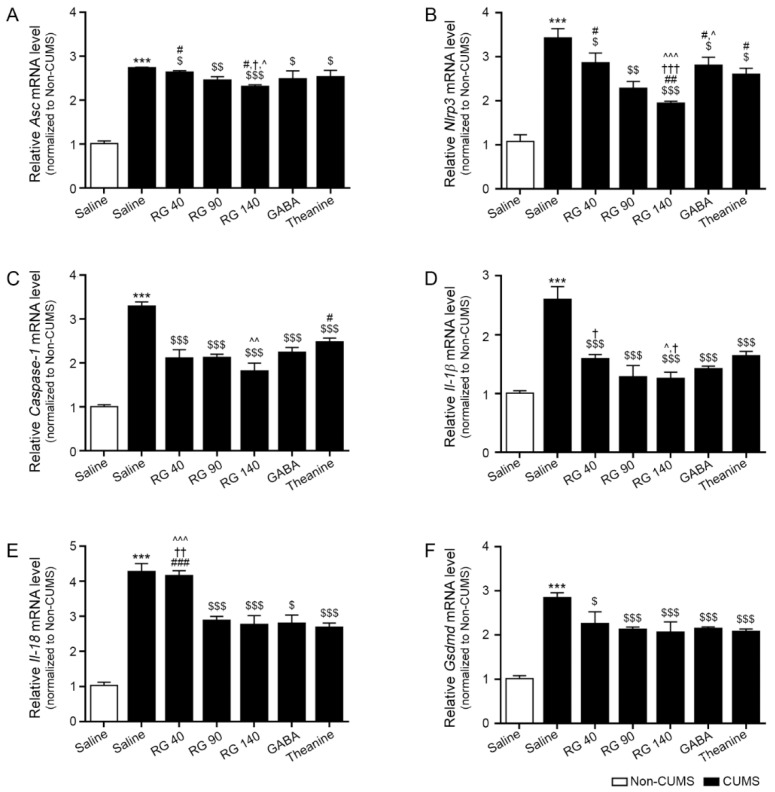
Reduction effects of RG on inflammasome and pyroptosis cytokines in the hypothalamus of CUMS mice model. (**A**–**F**) The mRNA levels of ASC (**A**), NLRP3 (**B**), Caspase-1 (**C**), IL-1β (**D**), IL-18 (**E**), and cell death mediated by GSDMD (**F**) were significantly upregulated in CUMS compared to those in the control but downregulated after RG, GABA, and theanine treatment. Data were normalized to *Actb,* and expression levels were reported relative to the control group using the comparative CT method. Data are presented as the mean ± SD (*n* = 3/group). ASC, apoptosis-associated speck-like protein containing a caspase recruitment domain; IL, interleukin; CUMS, chronic unpredictable mild stress; GABA, gamma-aminobutyric acid; GSDMD, gasdermin D; RG, rice germ with 30% GABA; NLRP3, NOD-like receptor family pyrin domain containing 3. ***, *p* < 0.05 vs. Non-CUMS; $, *p* < 0.05, $$, *p* < 0.01 and, $$$, *p* < 0.001 vs. CUMS; #, *p* < 0.05, ##, *p* < 0.01 and ###, *p* < 0.001 vs. CUMS/RG90; †, *p* < 0.05, ††, *p* < 0.01 and †††, *p* < 0.001 vs. CUMS/GABA; ^, *p* < 0.05, ^^, *p* < 0.01 and ^^^, *p* <0.001 vs. CUMS/Theanine.

CUMS exposure significantly increased apoptosis-associated speck-like protein containing a caspase recruitment domain (ASC) expression in the hypothalamus. It was decreased by oral administration of RG (40, 90, and 140 mg/kg), GABA, and theanine. The reduction effects of RG 140 mg/kg, GABA, and theanine were not significantly different (Figure 3A).

Following CUMS exposure, the expression of NLRP3 in the hypothalamus increased significantly. It was decreased by oral administration of RG (40, 90, and 140 mg/kg), GABA, and theanine. RG at 140 mg/kg had a greater inhibitory effect than GABA and theanine (Figure 3B).

Caspase-1 expression in the hypothalamus increased significantly following CUMS exposure. It was decreased by oral administration of RG (40, 90, and 140 mg/kg), GABA, and theanine. The inhibitory effect of RG at 140 mg/kg was greater than that of theanine (Figure 3C).

CUMS significantly increased the expression level of IL-1β in the hypothalamus. It was decreased by oral administration of RG (40, 90, and 140 mg/kg), GABA, and theanine. RG at 140 mg/kg had a greater inhibitory effect than GABA and theanine (Figure 3D).

CUMS significantly increased the expression of IL-18 in the hypothalamus. It was decreased by oral administration of RG (90 and 140 mg/kg), GABA, and theanine. The effects of RG at 90 and 140 mg/kg, GABA, and theanine on weight loss were not significantly different (Figure 3E).

CUMS significantly increased the expression of GSDMDs in the hypothalamus. It was decreased by oral administration of RG (40, 90, and 140 mg/kg), GABA, and theanine. The reduction effects of RG at 40, 90, and 140 mg/kg, GABA, and theanine were not significantly different (Figure 3F).

### 3.5. RB Attenuated Depressive-like Behavior in CUMS Mice

**Figure 4:**
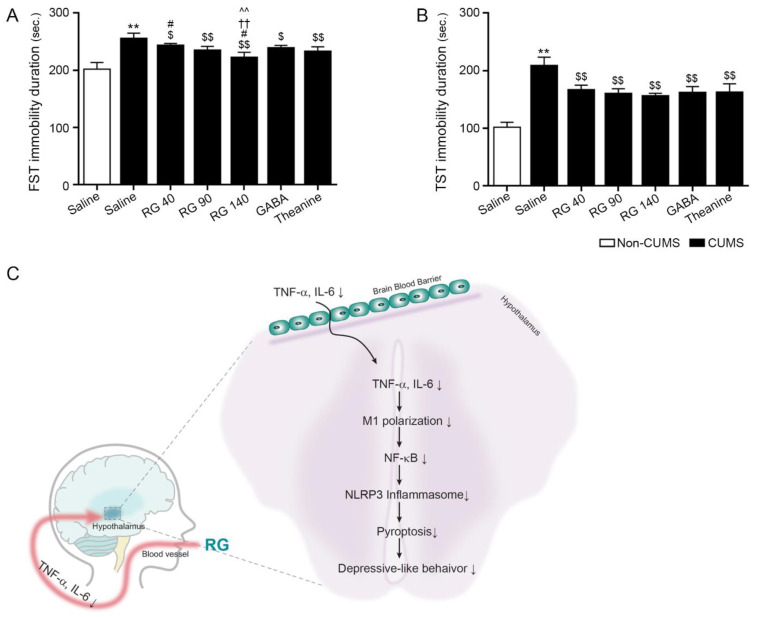
Effect of reducing anxiety-related depressive-like behavior by rice germ (RG) in the chronic unpredictable mild stress (CUMS) mice model. (**A**) The immobility duration of forced swimming test (FST) significantly increased in CUMS compared to that in the control; however, it decreased after treatment with RG, gamma-aminobutyric acid (GABA), and theanine. (**B**) The immobility duration of tail suspension test (TST) significantly increased in CUMS compared to that in the control; however, it decreased after treatment with RG, GABA, and theanine. Data are presented as the mean ± SD (*n* = 6/group). (**C**) Summary of this study. Due to RG intake, TNF- α and IL-6 in serum decreased (↓), and these cytokines pass through the BBB and acted on the hypothalamus. Cytokines that cross BBB (TNF- α and IL-6) suppressed (↓) TNF-α/IL-6, M1 polarization, NF-κB, and the NRLP3 inflammasome complex, which attenuated depressive-like behaviors. CUMS, chronic unpredictable mild stress; FST, forced swimming test; TST, tail suspension test; TNF-α, tumor necrotic factor-alpha; GABA, gamma-aminobutyric acid; IL-6, inerlukin-6; RG, rice germ with 30% GABA; NLRP3, NOD-like receptor family pyrin domain containing 3. **, *p* < 0.01 vs. Non-CUMS; $, *p* < 0.05, and $$, *p* < 0.01 vs. CUMS; #, *p* < 0.05 vs. CUMS/RG90; ††, *p* < 0.01 vs. CUMS/GABA; ^^, *p* < 0.01 vs. CUMS/Theanine.

Depressive-like behavior was evaluated using forced swim and tail suspension tests. The immobility duration during the forced swim test was increased by CUMS. It was attenuated by oral administration of RG (40, 90, and 140 mg/kg), GABA, and theanine. This attenuation was most significant by oral administration of RG at 140 mg/kg (Figure 4A).

CUMS increased the duration of immobility during the tail suspension test. It was decreased by oral administration of RG (40, 90, and 140 mg/kg), GABA, and theanine. The reduction effects of RG at 40, 90, and 140 mg/kg, GABA, and theanine were not significantly different (Figure 4B).

## 4. Discussion

Depression is characterized by various symptoms, including persistent low mood, anhedonia, loss of interest, and feelings of worthlessness [40]. Chronic stress induces mood, cognition, and memory abnormalities and leads to various brain diseases. The effects of stress on the brain have been evaluated using various animal models. Stress causes neuroinflammation and structural and functional alterations in neuronal networks [41,42].

The CUMS animal model, wherein animals are repeatedly exposed to varied unpredictable and uncontrollable stressors for days or weeks, is the most commonly used model [43,44,45,46,47]. CUMS is regarded as one of the most translationally relevant animal models for evaluating the pathophysiology of depression because of its reproducible neurochemical, neuroendocrine, and neuroinflammation outcomes [48,49]. Furthermore, CUMS also in-duces depressive behaviors; thus, it has been frequently used to evaluate the efficacy of antidepressants [49]. Thus, we evaluated the effect of RG on stress-induced depressive behaviors using the CUMS model.

Our study demonstrated that RG reduced expression levels of TNF-α and IL-6 in of serum CUMS mice. Additionally, RG reduced expression levels of TNF-α and IL-6 in the hypothalamic tissue.

Since GABA could not reach the brain directly, RG appeared to reduce pro-inflammatory cytokine release in peripheral blood across the BBB. Reduced levels of blood pro-inflammatory cytokines can attenuate neuroinflammation.

RG decreased hypothalamic M1 polarization and TNF-α and IL-6 levels. M1 polarization is reported to be elevated in MDD patients [50]. Furthermore, interferon-alpha-treated animals showed depressive-like behavior, which was accompanied by an increase in M1-type microglia [51].

Microglia are involved in the activation of the NLRP3 inflammasome complex in de-pression. The NLRP3 inflammasome complex is activated by two signaling pathways [52]. First, priming signals, such as N-κB induce NLRP3 upregulation. Following priming, the activating signal increases the binding between NLRP3 and the remaining inflammasome machinery, such as ASC and pro-caspase-1 [53]. Numerous studies have linked NLRP3 inflammasome complex or pyroptosis to depression or stress-induced depressive behavior [54]. Antidepressants decreased IL-1β and IL-18 levels in serum and suppressed NLRP3 expression in MDD patients and mice with stress-induced depression [55]. Chronic stress induces astrocyte loss in the hippocampus via pyroptosis [56]. Inhibition of the NLRP3 inflammasome complex by caspase-1 inhibitors, purinergic 2X7 receptor antagonists, or genetic deletion of NLRP3 showed a protective effect against stress-induced IL-β elevation [57,58,59]. In our investigation, RG decreased the hypothalamic levels of NLRP3, ASC, caspa-se-1, IL-1β, and IL-18. Moreover, depressive-like behaviors, which were evaluated using the forced swim test and tail suspension test, were also attenuated by RG in CUMS mice.

Conventional antidepressants, such as selective serotonin reuptake inhibitors, have been used previously [60]. However, antidepressant-treated patients with chronic inflammation or elevated baseline IL-6 and TNF levels had poor treatment responses [61,62,63]. Thus, regulating inflammation as an immune-targeted therapeutic for depression has been widely studied [64]. Stress is a well-known contributor to depression. However, there is no effective treatment for reducing stress as a preventive measure against depression. We thought that RG treatment could be an effective method for reducing stress-induced neuroinflammation because neuroinflammation is the primary mechanism of stress-induced depression.

In conclusion, RG inhibited multiple stress-induced neuroinflammation signaling pathways, which included decreasing TNF-α/IL-6, M1 polarization, NF-κB, and the NRLP3 inflammasome complex, which eventually attenuated depressive-like behaviors in CUMS mice (Figure 4C).

## Data Availability

Not applicable.

## Supplementary Materials

The following supporting information can be downloaded at: https://www.mdpi.com/article/10.3390/nu14245382/s1, Figure S1: Schematic diagram of CUMS procedure and animal experimental design; Figure S2: RG chemical analysis; Table S1: List of antibodies for ELISA and DAB staining; Table S2: List of primer for qRT-PCR; Table S3: Retention time of GABA standard and RG.
