# Supplementary material for: Rice Germ Ameliorated Chronic Unpredictable Mild Stress-Induced Depressive-like Behavior by Reducing Neuroinflammation
Source: Nutrients. 2022 Dec 18;14(24):5382. doi: 10.3390/nu14245382 (PMC9780988; doi:10.3390/nu14245382)
Supplement: Supplementary file 1 [file nutrients-14-05382-s001.zip › nutrients-2065749-supplementary.pdf]

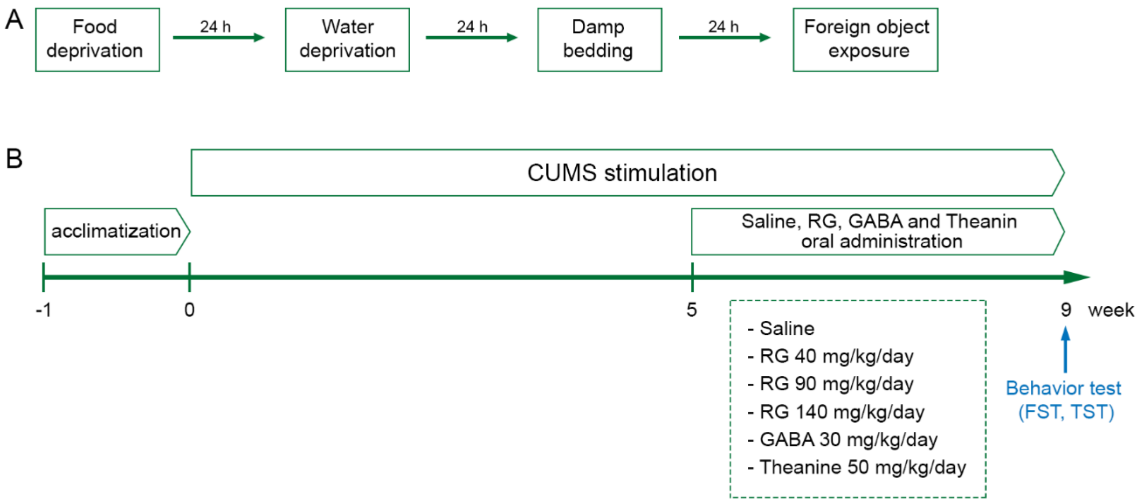

Figure S1. Schematic diagram of CUMS procedure and animal experimental design. (**A**) It is a schematic diagram showing stressors to induce CUMS. Each stressor was provided for 24 h. (**B**) After a one-week acclimatization period, the CUMS procedure was conducted for 5 weeks. After the CUMS procedure for 5 weeks, RG (40, 90 and 140 mg/kg/day), GABA (30 mg/kg/day), and theanine (50 mg/kg/day) were administered orally at the same time as the CUMS procedure for 4 weeks. Behavioral tests such as FST and TST were conducted at the last oral administration.

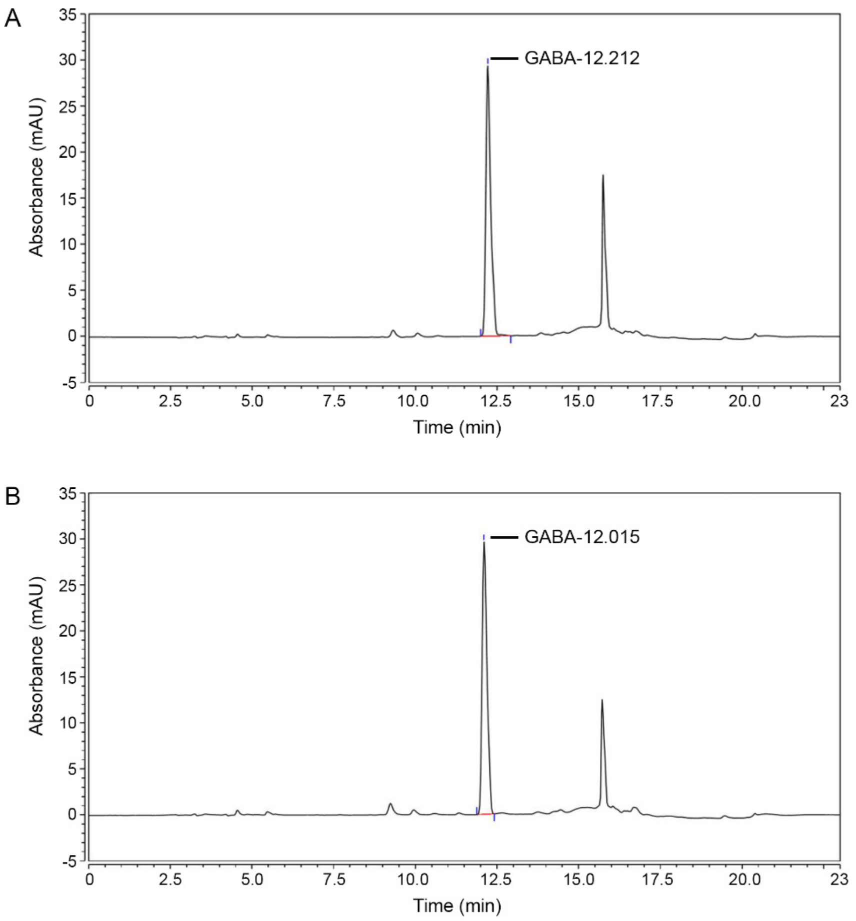

Figure S2. RG chemical analysis. (A and B) HPLC chromatograph of GABA standard (A) and GABA in RG (B).

Table S1. List of antibodies for ELISA and DAB staining.

Antibody	Company	Catalog no.	Dilution rate ELISA	Dilution rate DAB
TNF-α	Novus Biologicals	NBPI-19532	1:500 (0.2 µL anti-TNF-α : 100 µL PBS/well)	1:200 (0.5 µL anti-TNF-α : 100 µL normal serum/tissue)
IL-6	Santa Cruz Biotechnology	sc-57315	1:500 (0.2 µL anti-IL-6 : 100 µL PBS/well)	1:100 (1 µL anti-IL-6 : 100 µL normal serum/tissue)
CD206	Novus Biologicals	NBPI-90020	-	1:200 (0.5 µL anti-CD206 : 100 µL normal serum/tissue)
CD86	Santa Cruz Biotechnology	sc-19627	-	1:50 (2 µL anti-CD86 : 100 µL normal serum/tissue)
NF-κB	Cell Signaling Technology	8242S	-	1:200 (0.5 µL anti-NF-κB : 100 µL normal serum/tissue)

Table S2. List of primer for qRT-PCR.

Gene	Primers	
Actb	Forward	5'-CCGTAAAGACCTCTATGCCAAC-3′
	Reverse	5'-GCAGTAATCTCCTTCTGCATCC-3′
ASC	Forward	5′-GAAGGACAGTACCAGGCAGTTC -3′
	Reverse	5′-AGGATGGAACAAAGCTGAAGAG -3′
Caspase–1	Forward	5′-AGGACATCCTTCATCCTCAGAA -3′
	Reverse	5′-TTCTAAAGGGCAAAACTTGAGG -3′
GSDMD	Forward	5′-TCATGTGTCAACCTGTCAATCA -3′
	Reverse	5′-GACTTTGAAGGAGCCAAAACAC -3′
IL-1ß	Forward	5′-ACCAAGCAACGACAAAATACCT -3′
	Reverse	5′-CCGTCTTTCATTACACAGGACA -3′
IL-18	Forward	5′-GAAGACTCTTGCGTCAACTTCA -3′
	Reverse	5′-CTGATTCCAGGTCTCCATTTTC -3′
NLRP-3	Forward	5′-GAGGCCAAAAGGAAGGAGTATT -3′
	Reverse	5′-GGACCTCATTCTCTTGGATCAG -3′

Table S3. Retention time of GABA standard and RG.

Sample	Retention time (min)
GABA Standard	12.26 ± 0.092
RG	12.10 ± 0.001
